# 

**Published:** 1919-05

**Authors:** 


### ANNOUNCEMENTS

---

### ASSISTANTS WANTED

The Forsythe Dental Infirmary for children will furnish an opportunity for undergraduate dental students to engage in clinical practice during the summer months, for children in its infirmary, where every possible opportunity for getting experience under competent instructors in the various departments of children's rescue and preventive dentistry will be provided. It is a grand opportunity for students to get valuable experience in this special line of practice which is the future of dentistry.

The infirmary also offers a number of positions with good salaries for graduates for both part and whole time service. All the facilities and advantages of the institution are placed at the disposal of such operators, men and women graduates who contemplate specializing in children's work or orthodontia, will find this a grand opportunity for securing desirable preparations.

For full particulars write to Dr. Harold DeW. Cross, 140 Fenway, Boston, Mass.

---

### THE JOHN R. CALLAHAN MEMORIAL

At the December, 1918, meeting of The Ohio State Dental Society, a resolution was adopted to perpetuate the memory of the late John R. Callahan in recognition of his contributions to the science and art of dentistry and his unselfish devotion to its advancement throughout the many years of his professional life.

The Committee to which this Memorial was referred has decided on the following as the most appropriate and worthy of the approval of the profession.

1st—A Bronze Memorial to be placed in The Cincinnati General Hospital Grounds.

2nd—A Callahan Memorial Research Fund. The income from which to be awarded from time to time to the person making the best contribution to the Science and Art of Root Canal Problems. The fund and award to be under the direction of a committee perpetuated by The Ohio State Dental Society. The prize to be known as The John R. Callahan Award.

The sum necessary in the minds of the Committee to carry out the Memorial in its two phases should approximate $8,000, an amount that should speedily be raised in these times when the spirit of giving is universal.

Subscriptions to this fund by individuals or societies will be duly credited and should be forwarded to the Secretary-Treasurer of the fund.

*Committee*—T. Irving Way, Chairman, 52 Groton Building, Cincinnati; Henry E. Germann, Secretary-Treasurer, 719 Gwynne Building, Cincinnati; L. L. Barber, Toledo; Weston A. Price, Cleveland; L. E. Custer, Dayton; Edward C. Mills, Columbus.

---

### PREPAREDNESS LEAGUE NOTES

*Our Duty*—Early last autumn the President of the League wrote to different members of the Dental Corps in France, seeking information as to the condition of the dental profession in the devastated regions, but has been unable to get definite data except from Captain Blake Sears, who has, during his available time, been making a survey of those conditions which call for aid from the dental profession of America and Canada.

In a partial report Captain Sears states that there is a great need for help, and strongly approves of action in this direction. The matter was brought to the attention of Dr. Villian, who heartily endorses the movement and is anxious to collaborate with us. He was one of the organizers of a society in Paris to carry on this work. Funds were raised, which already have been exhausted, and the time is opportune for us to show our fraternal spirit by raising funds and supplying equipment for this society to place where it is most needed. Before this notice will be printed, we hope the movement will be well under way and some funds made available for this purpose.

*Home Service Work*—We are getting reports from different parts of the country of the excellent service our members are giving the worthy families of the Soldiers and Sailors. This is a most commendable object, and we sincerely trust that it will be continued so long as there is actual need. It is a splendid and practical way of demonstrating our readiness to help compensate for the sacrifices made by our boys.—J. W. BEACH, *President.*

---

### WE MUST KEEP FAITH

The American people were unitedly and earnestly back of Uncle Sam in the world war. They proved it at every point. They imposed no restrictions. They offered freely their lives, their services, their money. All in a degree unprecedented in American history. No demand was refused.

From every part of the country the response was immediate, forceful. No section slacked in any respect. Here and there there were individual slackers, but the cases were isolated, indifferently few. We were in effect unanimous.

America backed her ideals, her ambitions; stood for her rights, her honor, all that her forefathers had fought for and won.

And as we fought so must we finish.

We must keep faith with Uncle Sam, with the world at large, with the boys who won over there, with American welfare that is even yet at stake.

For few of us will care to stand before these returned soldier boys and confess that our only interest was in coming out of the scrimmage on top. That our interest in them ended when they won. That now that they're back it is up to them to shift for themselves. That our responsibility is ended.

We owe a further duty to America, a duty that is just as much to ourselves and our progeny. For America's future is keenly dependent upon the accomplishments of our industry and commercial enterprise within the next few years. We must buy the new Victory Bonds.

If you examine closely into your Liberty Bond, you will find that it stands alone as an investment. That nothing approaches it in absolute safety. That you can never lose by holding it. And that with every possibility of risk eliminated its interest return is really exceptional. Don't sell it.

